# Avian Influenza Virus Status and Maternal Antibodies in Nestling White Ibis (*Eudocimus albus*)

**DOI:** 10.3390/microorganisms9122468

**Published:** 2021-11-30

**Authors:** Katherine F. Christie, Rebecca L. Poulson, Julia Silva Seixas, Sonia M. Hernandez

**Affiliations:** 1 Odum School of Ecology, University of Georgia, Athens, GA 30602, USA; 2 Southeastern Cooperative Wildlife Disease Study, Department of Population Health, College of Veterinary Medicine, University of Georgia, Athens, GA 30602, USA; shernz@uga.edu; 3 Warnell School of Forestry and Natural Resources, University of Georgia, Athens, GA 30602, USA; julia.silvaseixas@uga.edu

**Keywords:** American white ibis, avian influenza virus, *Eudocimus albus*, maternal antibodies, nestling, wildlife

## Abstract

The White Ibis (*Eudocimus albus*), a nomadic wading bird, has increased its exploitation of urban habitats in South Florida, United States, and has recently established several urban breeding colonies. Certain characteristics of ibis ecology could position them in the natural cycle of the avian influenza virus (AIV). In fact, experimentally infected ibises were shown to be competent hosts for multiple AIV subtypes, and seroconversion to AIV has been documented in adult ibises in natural populations. However, the mechanisms of transmission and the timing of infection are unclear as we have yet to isolate AIV from a free-living ibis. To investigate the age-specific AIV dynamics of ibis, we captured nestlings (*n* = 115) weekly for 1–4 weeks from urban and natural settings in 2020 and 2021. We collected choanal/cloacal swabs for rRT-PCR and virus isolation, and plasma to screen for maternal AIV antibodies. AIV was not detected in any individual by virus isolation; however, maternal antibodies to AIV were detected in 95% of nestlings, with varying rates of catabolism. These results confirm that nestlings are afforded maternal antibodies from adults at rates reflective of higher adult seroprevalence than previously documented and that nestlings in breeding colonies may have some degree of protection and are unlikely to become infected with AIV.

## 1. Introduction

The White Ibis (hereafter, ibis; *Eudocimus albus*) is a nomadic wading bird common across the southeastern United States. Ibises usually breed in large, mixed species rookeries during the spring and early summer, with colonies sometimes housing as many as 60,000 – > 200,000 individuals in South Florida [1,2]. In the past 20 years, ibises have become common in urban areas, e.g., parks and landfills, foraging largely on anthropogenic food [3,4]. Recently, ibises have begun breeding in urban areas, altering conditions of ibis breeding such as community composition, diet, and stimuli (i.e., noise, light) [5,6,7]. Additionally, the reported expansion of the ibis’ range in the past four decades brings some ibis populations into areas important for migratory bird species, such as the Mississippi flyway [3]. These changes place both adult and nestling ibis at multiple important ecological interfaces with humans, urban wildlife, and wild avian populations, with the potential to alter pathogen dynamics in these groups.

Avian influenza viruses (AIV) are negative-sense single-stranded RNA viruses of the family Orthomyxoviridae. Viral subtypes are identified by their combination of hemagglutinin (HA) and neuraminidase (NA) surface glycoproteins, and AIV subtypes H1–H16 and N1–N9 have been detected in waterfowl and shorebirds [8]. AIV is maintained in some of these avian populations, which likely serve as the ultimate genetic reservoir from which influenza in other taxa arises [8]. Large congregations of wild birds play an important role in the transmission cycle of AIV, as demonstrated by the documented subtype reassortment events and poultry influenza outbreaks associated with the presence of migratory waterfowl [9,10]. In wild birds, transmission can occur through the fecal-oral route or be mediated through water [11,12]. While species in the orders Anseriformes and Charadriiformes are the most well-studied of the wild AIV reservoirs, other species may contribute to AIV maintenance and transmission in wildlife populations, with species-level variation in susceptibility, shedding rates, seasonal prevalence patterns, and transmission likelihood [8,10,13].

Aspects of ibis ecology such as aquatic association, nomadism, colonial breeding, and shared range with known AIV reservoirs like the mallard duck (*Anas platyrhynchos*) and ruddy turnstone (*Arenaria interpres morinella*) may make ibis susceptible to exposure and infection with AIV, but the degree to which they are involved in the annual cycle of AIV is unknown. Experimental infection of adult ibis has revealed that they are susceptible to and can shed multiple AIV subtypes, with an average shedding period of six days, which is comparable to many duck species [14]. However, AIV has not been isolated from any free-living ibis sampled in the winter, spring, or summer across multiple years in Florida [14]. AIV is often difficult to detect when sampling wild bird populations due to the short temporal window for shedding and the seasonal patterns exhibited by some species which affect prevalence and detection [8]. In free-living ibis, a high prevalence of AIV antibodies has been documented (average 70%) [14]. These data indicate that ibises are naturally infected and can shed AIV before seroconverting, though the timing and demographic profile of these infections in the wild and the role of this species in influencing AIV dynamics in their urban and natural habitats are unclear.

Nestling ibis may be vulnerable to infection due to immune naivety, as has been seen in other wading bird species, including species that share breeding colonies with the ibis. Many of the rarely isolated AIVs from the black crowned night heron (*Nycticorax nycticorax*) and snowy egret (*Egretta thula*) have originated from juveniles and nestlings in rookeries [15]. In some aquatic birds, such as mallards and some gull species, nestlings acquire maternal AIV antibodies, which have the potential to reduce AIV susceptibility among this young population, though most studies of wild nestling maternal AIV antibodies have been based on egg yolk antibody detection and thus do not address prevalence and persistence in nestlings [16,17,18]. Determining whether ibis nestlings are infected with AIV, considering the potential age-specific immunological characteristics, is crucial to defining the timeline of AIV infection in this species, which differs from other known reservoirs and may potentially affect vulnerable sympatric birds. Additionally, this information is necessary for detecting the shifts in prevalence that may occur if urban ibis breeding colonies become more common.

The objectives of this study were to determine whether nestling ibises were infected with AIV and whether pathogen dynamics in this age group were influenced by the landscape (urban versus natural) of the breeding colony. Through weekly sampling of ibis nestlings in both urban and natural colonies in South Florida, United States in 2020 and 2021, we detected no AIV in nestlings from either landscape but found a high prevalence of maternal antibodies across sites and years.

## 2. Materials and Methods

**Figure 1:**
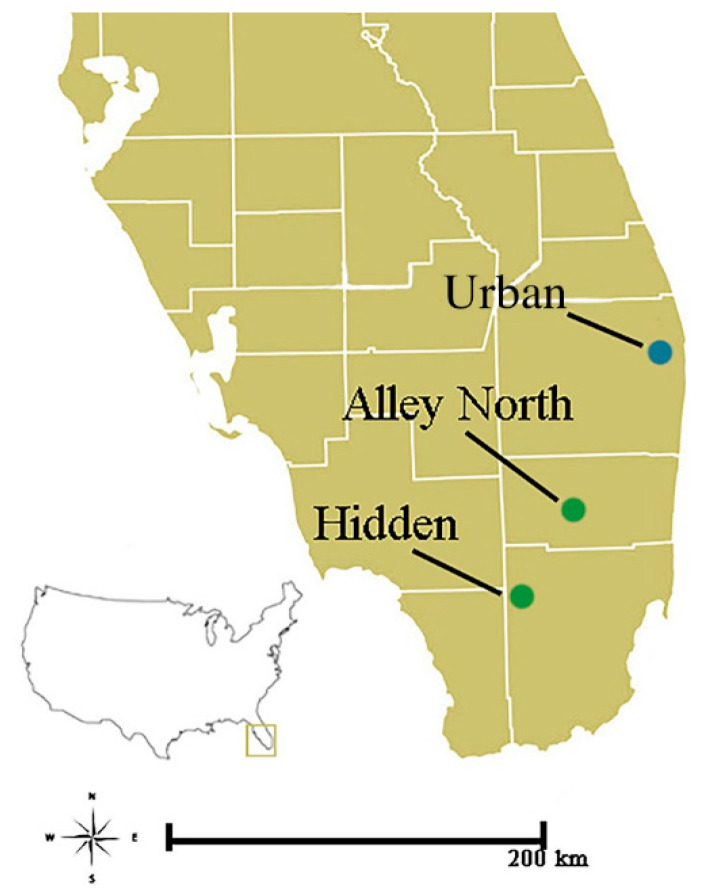
White Ibis (*Eudocimus albus*) rookery locations from which nestlings were sampled. The urban rookery (blue), located in Palm Beach County, FL, was sampled both in 2020 and 2021; the two natural rookeries (green), located in Broward and Miami-Dade Counties, were sampled only in 2021.

Nestling ibises were sampled in breeding colonies in urban and natural areas. The urban colony was located in Palm Beach County, Florida, the third most populated county in the state, and was made up of three adjacent islands in a water body within a golf community (26.825, −80.149444). Samples were also collected from two natural-area colonies in the Everglades Water Conservation Area 3A and Frances S. Taylor Wildlife Management Area (Alley North: 26.191096, −80.523351; Hidden: 25.776278, −80.840643) in neighboring Broward and Miami-Dade counties (Figure 1).

Other aquatic bird species cohabitating with ibis in both urban and natural rookeries included the snowy egret, great egret (*Ardea alba*), black crowned night heron, and glossy ibis (*Plegadis falcinellus*); though infrequent, AIV has been detected from each of these species [15,19,20].

Samples were collected from the urban rookery between April–July 2020 and 2021, and from the natural rookeries between March - June 2021. Urban and natural sites were visited at least once weekly, with new nests marked and checked at each visit. Upon hatching and every week thereafter, one nestling from each nest (urban, *n* = 77; natural, *n* = 38) was captured by hand until the nestling was too mobile for capture, most commonly occurring at between two and three weeks of age. Sampled nestlings were banded and marked with a water-based dye to ensure consistency across weeks. Age at time of sampling ranged from 1 to 25 days old. Similar procedures were followed at the natural rookery site. Weekly nest checks at the natural sites began only after nestling hatching, not at egg-laying; as such, nestlings tended to be captured for the first time at a later age than those in the urban rookery (in 2021, average of the first capture of 3.8 days in urban rookery; 9.6 days in natural rookery). At the urban rookery in both 2020 and 2021, average clutch size was 2.52 and average fledging rates were 1.98 (2020) and 1.86 (2021). Average clutch sizes at the natural sites (Alley North and Hidden) were 2.10 and 2.62 with average fledging rates of 1.69 and 1.51, respectively.

Each week, a sterile swab was used to collect a paired choanal/cloacal (CH/CL) sample which was stored in chilled viral transport media containing 2 mL Brain Heart Infusion (Becton Dickinson and Co. Sparks, MD, USA) supplemented with penicillin G (1000 units/mL), streptomycin (1 mg/mL), kanamycin (0.5 mg/mL), gentamicin (0.25 mg/mL), and amphotericin B (0.025 mg/mL) (Sigma Chemical Company, St. Louis, MO, USA). Blood was collected from the jugular or leg vein of each individual, not exceeding 1% of the body weight, and stored in tubes coated in lithium heparin anticoagulant. Blood was centrifuged for 10 min at 1350× *g* within six hours of collection and plasma was aliquoted before freezing. All samples were maintained at 4 °C in the field for no more than five hours and then either refrigerated at 4 °C (<5 days) and shipped overnight or frozen in liquid nitrogen until transport to the Southeastern Cooperative Wildlife Disease Study (SCWDS) in Athens, Georgia, where they were stored at −80 °C until processing.

AIV prevalence estimates were based on virus isolation (VI) as previously described [21]. Briefly, CH/CL swabs were thawed, vortexed, and centrifuged at 1500× *g* for 15 min. Supernatant from each sample was inoculated (0.33 mL per egg) into three 9–11 day-old embryonated chicken eggs via the allantoic route. Eggs were incubated at 37 °C for five days; amnio-allantoic fluid was harvested from each egg and tested via hemagglutination assay using 0.5% chicken red blood cells [22].

Viral RNA extraction was carried out on all swab samples using MagMAX-96 AI/ND Viral RNA Isolation kit (Ambion, Austin, TX, USA) on the Thermo Electron KingFisher magnetic particle processor (Thermo Electron Corporation, Waltham, MA, USA) [23]. All extracted RNA was analyzed using real-time reverse transcriptase PCR (rRT-PCR) targeting the matrix gene to confirm AIV presence or absence [24]; a cycle-threshold (Ct) value of <45 cycles was considered positive for AIV RNA.

Plasma samples were screened using a commercial blocking enzyme-linked immunosorbent assay (bELISA; FlockCheck AI MultiS-Screen Antibody Test Kit, IDEXX Laboratories, Westbrook, ME, USA) as per the manufacturer’s instructions. The use of plasma with this commercial bELISA kit has not been validated, however, plasma was used to establish the seroprevalence of adult ibis in an analogous study [14] and plasma has been reported as a suitable sample for bELISA for the detection of avian IAV antibodies [25]. Further, an investigation into the validity of detecting IAV antibodies in bELISA assays using plasma versus serum has shown that heparin plasma yields statistically equal results to serum in paired sample studies [26]. In our study, each sample was tested in duplicate on separate plates, and absorbance at 650 nm was measured using an EMax Plus microplate reader (Molecular Devices, San Jose, CA, USA). Plasma samples with sample-to-negative control (S/N) absorbance values ≤0.70 were considered positive for antibodies to AIV [27,28]. The full table of samples and results for all laboratory analyses are included in Table S1, linked in the Supplementary Materials section.

Data were visualized in RStudio using the ′ggplot2′ package [29].

Rookery access was granted under access permit PER 11-464. Animal handling and laboratory analysis permissions were granted by federal scientific collecting permit MB53675D-0, Florida scientific collecting permit LSSC-11-00119H, and UGIACUC Animal Use Permits A2019 10-009-Y1-A0 and A2019 04-001-A3.

## 3. Results

In total, 271 CH/CL swab samples were screened, representing a longitudinal sampling of 115 ibis nestlings (urban, 2020 *n* = 36; urban, 2021 *n* = 41; natural, 2021 *n* = 38) in their first weeks of life. No AIV were isolated from any samples from the urban nor natural ibis rookeries in 2020 or 2021, at any time point. Two samples yielded an rRT-PCR Ct value below the 45-cycle threshold, one from a 15-day old urban rookery nestling in 2020 (Ct 43.98) and one from 6-day old natural rookery nestling sampled in 2021 yielded (Ct 33.98); AIV viral RNA was not detected in any other samples (Table 1).

**Figure 2:**
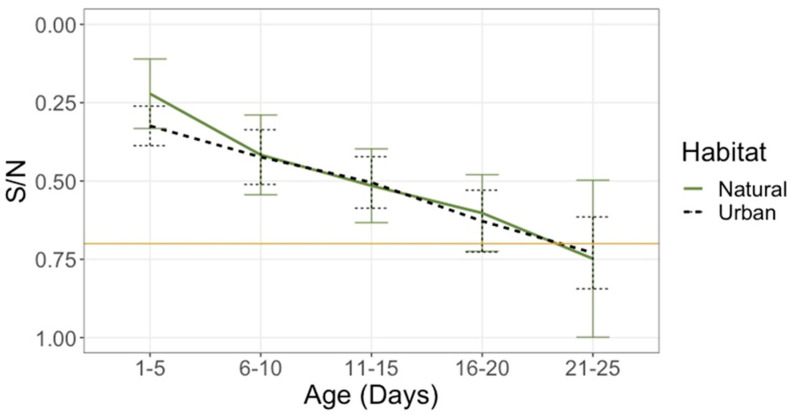
The persistence of avian influenza virus (AIV) antibodies in ibis nestlings. Average sample to negative (S/N) ratio for plasma samples (urban *n* = 1 63; natural *n* = 66) from 2020 and 2021 in a south Florida urban rookery (black dotted) and from the 2021 breeding season in natural rookeries (green solid). Error bars represent a standard error calculation. The higher S/N ratio reflects the lower concentration of AIV antibodies.

Based on bELISA results, 95% (*n* = 101) of nestlings sampled from both urban and natural wetlands were positive for AIV nucleoprotein (NP) antibodies at some point during sampling. Seroprevalence was high across sites and years, with prevalence at the urban rookery of 100% in 2020 (*n* = 22 nestlings) and 98% in 2021 (*n* = 41), and 89% (*n* = 38) at the natural rookery. In addition to the site-dependent variation in average age at first capture, capture frequency also varied across sites and years due to the terrain, with 2.14 average captures per bird in the urban rookery in 2020 and 2.74 in 2021, and 1.74 in the natural rookery in 2021. Nearly all serially sampled birds displayed a pattern consistent with the presence and decay of maternal antibodies, with average S/N ratios increasing with age (Figure 2). The overall seroprevalence of all samples across urban and natural habitats was 83% (*n* = 229), with some later samples from previously antibody-positive individuals showing S/N ratios above the positivity threshold. Plasma samples from a subset of birds (*n* = 14) from 2020 were degraded upon arrival at SCWDS and removed from the analysis.

The persistence of NP antibodies differed between nestlings, with S/N ratios varying within the same age group. For example, the S/N ratio of one antibody-positive urban nestling increased to >0.7 by eight days of age, while another was still antibody-positive at 22 days of age. Given that many nestlings were still antibody-positive at their final sampling (70%, *n* = 101, age range = 1–25 days), the average age at which these birds catabolize their maternal antibodies could not be determined, though it may be greater than 2–3 weeks.

## 4. Discussion

Ibis nestlings sampled throughout their first three weeks of life in urban and natural colonies in Florida were not found to be infected with AIV. Ibis nestlings in both environments had a high seroprevalence (95%, *n* = 101) and persistence of maternal antibodies, which, paired with the absence of AIV isolation, suggests that they may be protected from AIV infection. Although the apparent prevalence of maternal antibodies was lower in nestlings from the natural colonies, we cannot state that this difference was significant due to the variation in nest tracking scheme and rookery conditions between the urban and natural colony which led to a higher age of the first capture and lower recapture likelihood at the natural rookeries. Additionally, we were only able to collect samples from the natural colony during 2021 due to restrictions during the start of the COVID-19 pandemic, and thus only have one year of data to compare across habitats.

These results indicate that the AIV exposure of wild adult egg-laying female ibis was nearly 100%, as they must have had circulating antibodies at the time of egg development to yield the prevalence of nestling antibodies detected. This is higher than the antibody prevalence previously measured in adult ibis [14], which may be due to a previously undetected seasonal or demographic pattern in antibody prevalence. The ibis breeding season (variable, but typically from February to June) may be the period where transmission and adult infections are most common, leading to high antibody prevalence and the observed maternal antibody transference. This part of the year has rarely been included in adult sampling efforts, as even highly urbanized ibis still typically nest in natural colonies, where they are difficult to capture. Alternatively, there may be a sex bias in adult antibody prevalence, with females having higher antibody levels that are then passed to offspring, though this has not been documented to date.

The AIV seroprevalence estimates in this study are higher than those reported in other wild aquatic birds, though studies of this type are rare. In the yellow-legged gull (*Larus michahellis*) and mallard, egg yolk AIV antibody prevalence, as determined by NP bELISA, is lower than adult antibody prevalence detected at the same time and location, indicating that not all antibody-positive females pass antibodies to their offspring [17,18]. Maternal AIV antibody passage is variable among aquatic birds; adult northeastern Pacific red knots (*Calidris canutus roselaari*) had an estimated AIV antibody prevalence of >90%, but no sampled nestlings (<2 weeks old, *n* = 16) had detectible antibodies based on bELISA analysis [30]. The mothers of the ibis nestlings in this study transferred antibodies to their offspring, and in many cases, these antibodies persisted for several weeks. No nestlings were infected with AIV during these first 23–25 days of life, which may indicate that ibises have a relatively high investment in humoral immunity to AIV and that the transfer of maternal antibodies is an important part of their relationship with this pathogen.

Our objective was to determine whether ibis are commonly infected with AIV as nestlings, which could explain the high seroprevalence of AIV antibodies in adult ibis [14]. Our VI and serology results indicate that, in the Florida ibis system, it is unlikely that ibises become infected as young nestlings. A repeated serological sampling of nestlings provides a glimpse into the process of maternal antibody decay in wild birds; further analysis of a species such as an ibis with high antibody prevalence could elucidate the potential timing and effects of maternal antibody protection in other colonial aquatic birds.

The timing of AIV infections in the ibis and other understudied aquatic birds is of interest because it often differs from well-studied waterfowl AIV cycles and because of the nomadic nature and close association with humans of some of these species. Mallard ducks, the model species for AIV in wild birds, have a distinct infection timeline that includes a high prevalence of infection during fall, especially among juveniles, with a predictable cycle from year to year [19,31,32,33,34]. In ibis, as with many other aquatic birds, a distinctive schedule of infection has yet to be determined, due to the low infection rates found in natural populations [11,13,14,15,19,20]. These understudied aquatic species may have the opportunity to be infected by and transmit to other AIV hosts, including mallards and other ducks, and shorebirds. In the case of the ibis, the species’ large range intersects with known AIV hosts in both urban and natural habitat across the southeastern United States including Louisiana, a state with large waterfowl aggregations, where ibis could potentially be influencing pathogen dynamics along the Mississippi flyway [8]. As such, understanding the AIV dynamics of non-model species remains important to understanding how AIV cycles in wild birds in critical habitats and how this may change as urbanization alters the way host species live and form communities. In particular, prolific nomadic ibis populations may create a bridge between urban and natural areas, and the addition of breeding colonies and immunologically vulnerable young ibis to the urban environment could shift the potential for transmission. This makes the comparison of pathogen dynamics in urban and natural aquatic bird breeding environments important for understanding risks to public health and wild avian populations.

To better understand AIV infection dynamics in understudied aquatic bird populations, species such as the ibis should be subject to surveillance efforts soon after fledging, as the current study indicates that some species may be granted some degree of protection from maternally derived antibodies during their earlier stages of life. Additionally, serologic and virologic sampling of adult wading birds at mixed species breeding colonies could reveal whether the gathering at these sites leads to increased AIV prevalence, which could explain the high levels of maternal antibodies found in ibis nestlings. Additionally, the AIV subtype specificity of the maternal antibodies acquired by ibis nestlings and whether they are protective remains unknown, and these details may be useful in establishing the vulnerability of wading bird nestlings in rookeries, as there are few other studies documenting maternal antibody acquisition and decay in wild birds. As it stands, this study begins to fill epidemiological gaps and provides guidance for continued work in understanding host-pathogen relationships and the role of maternal antibody transference in the ibis and other aquatic birds whose AIV status is poorly understood.

## Figures and Tables

**Table 1:** Total swab samples collected for avian influenza virus (AIV) isolation and real-time reverse transcriptase-polymerase chain reaction (rRT-PCR) screening from ibis nestlings in urban rookeries in 2020 and urban and natural rookeries in 2021. Nestlings were sampled weekly from hatch until approximately 3 weeks of age.

Collection Year	Habitat Type	Number of Birds (n Samples)	Number of Samples AIV Positive by Virus Isolation/Positive by rRT-PCR (Ct ^a^ value)
2020	Urban	36 (94)	0/1 (43.98)
2021	Urban	42 (114)	0/0 (n/a ^b^)
2021	Natural	38 (61)	0/1 (33.98)

^a^ Cycle-threshold (Ct), ^b^ Not applicable (n/a).

## Data Availability

The data presented in this study are available in the Supplemental Table S1: Ibis Nestling Sample Inventory and Laboratory Results.

## Supplementary Materials

The following are available online at https://www.mdpi.com/article/10.3390/microorganisms9122468/s1, Table S1: Ibis Nestling Sample Inventory and Laboratory Results.
