# Supplementary material for: Avian Influenza Virus Status and Maternal Antibodies in Nestling White Ibis (Eudocimus albus)
Source: Microorganisms. 2021 Nov 30;9(12):2468. doi: 10.3390/microorganisms9122468 (PMC8707160; doi:10.3390/microorganisms9122468)
Supplement: Supplementary file 1 [file microorganisms-09-02468-s001.zip › microorganisms-1424987-supplementary.pdf]

Collection information				Serological Screening		Virc
Year	Habitat	Ibis Field ID[++]	Age (days)	Plasma Sample ID	S/N[*]	Swab Sample ID
2020	Urban	1	2	70	0.078	IBIS20-047
2020	Urban	1	9	69	0.325	IBIS20-052
2020	Urban	1	15	65	0.529	IBIS20-062
2020	Urban	1	23	44	0.731	IBIS20-072
2020	Urban	2	1	46	0.059	IBIS20-049
2020	Urban	2	8	45	0.221	IBIS20-058
2020	Urban	2	15	5	0.214	IBIS20-068
2020	Urban	3	1	NS[A]	NA[B]	IBIS20-061
2020	Urban	3	8	90	0.327	IBIS20-056
2020	Urban	4	1	91	0.217	IBIS20-048
2020	Urban	5	2	71	0.118	IBIS20-050
2020	Urban	5	8	68	0.356	IBIS20-059
2020	Urban	5	15	58	0.502	IBIS20-069
2020	Urban	6	2	59	0.062	IBIS20-051
2020	Urban	6	10	61	0.313	IBIS20-060
2020	Urban	7	2	62	0.049	IBIS20-066
2020	Urban	7	11	1	0.300	IBIS20-065
2020	Urban	7	17	89	0.366	IBIS20-070
2020	Urban	8	1	67	0.079	IBIS20-053
2020	Urban	9	5	66	0.131	IBIS20-057
2020	Urban	9	12	49	0.303	IBIS20-067
2020	Urban	9	24	28	0.568	IBIS20-083
2020	Urban	10	2	60	0.097	IBIS20-063
2020	Urban	10	11	51	0.425	IBIS20-073
2020	Urban	10	22	26	0.619	IBIS20-086
2020	Urban	11	5	88	0.469	IBIS20-064
2020	Urban	11	14	73	0.623	IBIS20-074
2020	Urban	11	22	72	0.680	IBIS20-084
2020	Urban	12	3	63	0.056	IBIS20-054
2020	Urban	12	12	48	0.269	IBIS20-071
2020	Urban	12	20	56	0.503	IBIS20-085
2020	Urban	13	1	55	0.093	IBIS20-055
2020	Urban	14	1	52	0.078	IBIS20-081
2020	Urban	14	8	53	0.356	IBIS20-093
2020	Urban	14	17	NS	NA	IBIS20-010
2020	Urban	14	21	NS	NA	IBIS20-011
2020	Urban	15	4	64	0.130	IBIS20-078
2020	Urban	15	12	15	0.372	IBIS20-090
2020	Urban	15	18	NS	NA	IBIS20-006
2020	Urban	16	7	50	0.091	IBIS20-075
2020	Urban	16	15	7	0.705	IBIS20-087
2020	Urban	16	21	NS	NA	IBIS20-002
2020	Urban	17	4	74	0.212	IBIS20-076

2020	Urban	17	12	75	0.403	IBIS20-088
2020	Urban	17	18	NS	NA	IBIS20-003
2020	Urban	17	25	NS	NA	IBIS20-004
2020	Urban	18	4	47	0.049	IBIS20-077
2020	Urban	18	12	54	0.122	IBIS20-089
2020	Urban	18	18	NS	NA	IBIS20-005
2020	Urban	19	3	84	0.175	IBIS20-079
2020	Urban	19	11	76	0.428	IBIS20-091
2020	Urban	19	20	NS	NA	IBIS20-007
2020	Urban	19	24	NS	NA	IBIS20-008
2020	Urban	20	4	57	0.081	IBIS20-080
2020	Urban	20	12	3	0.268	IBIS20-092
2020	Urban	20	21	NS	NA	IBIS20-009
2020	Urban	21	1	82	0.052	IBIS20-082
2020	Urban	21	8	6	0.261	IBIS20-094
2020	Urban	21	17	NS	NA	IBIS20-001
2020	Urban	22	11	NS	NA	IBIS20-038
2020	Urban	23	13	NS	NA	IBIS20-039
2020	Urban	24	3	NS	NA	IBIS20-012
2020	Urban	25	3	NS	NA	IBIS20-015
2020	Urban	25	11	NS	NA	IBIS20-014
2020	Urban	26	2	NS	NA	IBIS20-013
2020	Urban	26	12	NS	NA	IBIS20-016
2020	Urban	27	2	NS	NA	IBIS20-036
2020	Urban	27	14	NS	NA	IBIS20-037
2020	Urban	28	5	NS	NA	IBIS20-017
2020	Urban	28	10	NS	NA	IBIS20-018
2020	Urban	28	17	NS	NA	IBIS20-019
2020	Urban	29	2	NS	NA	IBIS20-029
2020	Urban	29	11	NS	NA	IBIS20-030
2020	Urban	29	18	NS	NA	IBIS20-031
2020	Urban	30	2	NS	NA	IBIS20-020
2020	Urban	30	7	NS	NA	IBIS20-021
2020	Urban	30	14	NS	NA	IBIS20-022
2020	Urban	30	21	NS	NA	IBIS20-023
2020	Urban	31	3	NS	NA	IBIS20-024
2020	Urban	31	8	NS	NA	IBIS20-025
2020	Urban	32	2	NS	NA	IBIS20-026
2020	Urban	32	7	NS	NA	IBIS20-027
2020	Urban	32	14	NS	NA	IBIS20-028
2020	Urban	33	1	NS	NA	IBIS20-044
2020	Urban	33	6	NS	NA	IBIS20-045
2020	Urban	33	13	NS	NA	IBIS20-046
2020	Urban	34	2	NS	NA	IBIS20-032
2020	Urban	34	7	NS	NA	IBIS20-033
2020	Urban	34	14	NS	NA	IBIS20-034
2020	Urban	34	21	30	0.102	IBIS20-035

2020	Urban	35	8	NS	NA	IBIS20-042
2020	Urban	35	15	NS	NA	IBIS20-043
2020	Urban	36	2	NS	NA	IBIS20-040
2020	Urban	36	9	NS	NA	IBIS20-041
2021	Urban	101	1	23	0.249	NS
2021	Urban	101	8	94	0.721	60
2021	Urban	101	16	133	0.952	63
2021	Urban	101	23	165	1.003	126
2021	Urban	102	3	21	0.146	13
2021	Urban	102	12	121	0.609	80
2021	Urban	102	21	152	0.794	102
2021	Urban	103	2	22	0.136	12
2021	Urban	103	9	99	0.437	47
2021	Urban	103	21	182	0.766	105
2021	Urban	104	2	93	0.327	61
2021	Urban	104	8	114	0.681	78
2021	Urban	104	14	105	0.771	77
2021	Urban	104	22	235	0.803	131
2021	Urban	105	2	95	0.281	59
2021	Urban	105	10	128	0.558	70
2021	Urban	105	17	181	0.770	106
2021	Urban	105	24	NS	NA	153
2021	Urban	106	1	97	0.436	52
2021	Urban	106	11	150	0.839	100
2021	Urban	107	2	103	0.378	48
2021	Urban	107	12	151	0.722	122
2021	Urban	107	18	205	0.776	140
2021	Urban	108	2	96	0.420	49
2021	Urban	109	2	100	0.277	53
2021	Urban	109	12	153	0.643	121
2021	Urban	109	18	204	0.654	141
2021	Urban	110	3	98	0.241	50
2021	Urban	111	1	101	0.265	51
2021	Urban	112	1	117	0.401	71
2021	Urban	112	7	106	0.487	72
2021	Urban	112	15	233	0.656	132
2021	Urban	112	23	261	0.864	171
2021	Urban	113	1	115	0.481	74
2021	Urban	113	9	148	0.487	96
2021	Urban	113	18	247	0.658	NS
2021	Urban	114	2	116	0.493	69
2021	Urban	115	1	119	0.381	84
2021	Urban	115	7	189	0.606	119
2021	Urban	115	13	177	0.854	115
2021	Urban	115	20	222	0.831	160
2021	Urban	116	4	124	0.788	82
2021	Urban	116	10	190	0.693	120

2021	Urban	116	16	172	0.824	111
2021	Urban	117	1	125	0.393	85
2021	Urban	117	7	191	0.351	118
2021	Urban	117	13	175	0.473	110
2021	Urban	117	20	226	0.568	164
2021	Urban	118	1	123	0.415	81
2021	Urban	118	10	160	0.447	125
2021	Urban	118	15	202	0.524	142
2021	Urban	118	21	255	0.713	166
2021	Urban	119	1	120	0.467	86
2021	Urban	119	11	163	0.567	104
2021	Urban	119	17	213	0.656	150
2021	Urban	120	4	118	0.460	83
2021	Urban	120	13	183	0.384	123
2021	Urban	120	18	238	0.416	138
2021	Urban	121	4	131	0.565	64
2021	Urban	122	3	129	0.614	62
2021	Urban	122	10	157	0.745	128
2021	Urban	122	16	214	0.761	151
2021	Urban	123	3	130	0.625	65
2021	Urban	123	10	162	0.726	130
2021	Urban	123	16	NS	NA	148
2021	Urban	123	25	279	0.925	178
2021	Urban	124	3	109	0.425	66
2021	Urban	124	11	201	0.519	136
2021	Urban	125	4	104	0.974	67
2021	Urban	126	5	113	0.313	79
2021	Urban	126	13	232	0.340	133
2021	Urban	127	3	108	0.403	68
2021	Urban	127	11	196	0.448	137
2021	Urban	127	19	264	0.445	170
2021	Urban	128	3	112	0.345	76
2021	Urban	128	11	199	0.563	135
2021	Urban	128	19	260	0.615	168
2021	Urban	129	4	107	0.356	75
2021	Urban	129	12	198	0.512	134
2021	Urban	129	20	262	0.741	169
2021	Urban	130	4	144	0.364	93
2021	Urban	130	10	179	0.462	114
2021	Urban	130	17	223	0.429	161
2021	Urban	131	2	188	0.350	117
2021	Urban	131	8	176	0.715	112
2021	Urban	131	15	230	0.603	163
2021	Urban	132	4	192	0.407	94
2021	Urban	132	7	156	0.331	NS
2021	Urban	132	10	173	0.399	116
2021	Urban	132	17	224	0.485	162

2021	Urban	133	3	146	0.329	97
2021	Urban	133	11	209	0.254	146
2021	Urban	133	19	267	0.371	176
2021	Urban	134	7	147	0.323	98
2021	Urban	134	15	207	0.594	147
2021	Urban	134	23	268	1.009	173
2021	Urban	135	2	185	0.263	99
2021	Urban	135	10	244	0.476	143
2021	Urban	135	18	273	0.760	174
2021	Urban	136	2	149	0.476	95
2021	Urban	136	10	206	0.498	144
2021	Urban	136	18	270	0.519	172
2021	Urban	136B	9	237	0.237	NS
2021	Urban	136B	11	208	0.287	145
2021	Urban	137	7	NS	NA	124
2021	Urban	137	10	174	0.282	113
2021	Urban	137	17	227	0.288	159
2021	Urban	138	6	184	0.467	103
2021	Urban	138	11	241	0.347	139
2021	Urban	138	17	258	0.625	165
2021	Urban	139	6	158	0.375	101
2021	Urban	139	13	217	0.407	149
2021	Urban	139	17	256	0.558	167
2021	Urban	139	22	278	0.613	177
2021	Urban	140	8	168	0.403	129
2021	Urban	140	14	210	0.472	152
2021	Urban	140	21	269	0.484	175
2021	Urban	141	5	164	0.512	127
2021	Natural	E1	4	32	0.174	NS
2021	Natural	E1	11	24	0.405	27
2021	Natural	E2	5	29	0.158	NS
2021	Natural	E2	12	26	0.399	26
2021	Natural	E2	20	43	0.484	22
2021	Natural	E3	3	28	0.201	NS
2021	Natural	E4	1	30	0.161	NS
2021	Natural	E5	4	35	0.124	NS
2021	Natural	E5	11	37	0.195	24
2021	Natural	E5	19	55	0.216	NS
2021	Natural	E7	2	27	0.183	NS
2021	Natural	E7	9	40	0.349	25
2021	Natural	E7	17	44	0.450	20
2021	Natural	E8	11	14	0.443	28
2021	Natural	E8	19	51	0.259	NS
2021	Natural	E9	1	38	0.107	32
2021	Natural	E9	9	46	0.318	14
2021	Natural	E9	15	57	0.399	27
2021	Natural	E10	1	41	0.295	31

2021	Natural	E10	9	42	0.400	21
2021	Natural	E10	15	69	0.543	33
2021	Natural	E11	1	36	0.170	30
2021	Natural	E11	9	45	0.283	18
2021	Natural	E12	1	33	0.146	23
2021	Natural	E12	9	47	0.387	15
2021	Natural	E12	15	68	0.512	41
2021	Natural	E13	2	25	0.171	29
2021	Natural	E13	10	50	0.420	16
2021	Natural	E13	16	58	0.444	39
2021	Natural	E13	23	6	0.976	7
2021	Natural	E14	12	NS	NA	17
2021	Natural	E14	18	74	1.047	43
2021	Natural	E15	17	NS	NA	19
2021	Natural	E15	23	60	0.529	38
2021	Natural	E16	10	59	0.646	35
2021	Natural	E17	10	66	0.408	42
2021	Natural	E18	11	72	0.501	34
2021	Natural	E19	10	61	0.379	36
2021	Natural	E20	15	67	0.467	40
2021	Natural	E20	22	1	0.744	6
2021	Natural	E21	10	8	0.429	8
2021	Natural	E22	10	2	0.836	3
2021	Natural	E23	11	9	0.259	1
2021	Natural	E24	10	10	0.231	2
2021	Natural	E25	11	5	0.246	11
2021	Natural	E26	11	NS	NA	4
2021	Natural	E27	8	3	0.383	5
2021	Natural	E28	11	86	0.527	58
2021	Natural	E28	18	134	0.809	90
2021	Natural	E29	12	82	0.617	44
2021	Natural	E29	19	142	0.732	87
2021	Natural	E30	13	81	0.500	57
2021	Natural	E31	6	88	0.501	46
2021	Natural	E31	13	135	0.620	91
2021	Natural	E31	20	170	0.667	108
2021	Natural	E32	5	83	0.414	54
2021	Natural	E32	12	139	0.616	92
2021	Natural	E32	19	171	0.723	109
2021	Natural	E33	5	91	0.502	55
2021	Natural	E33	12	137	0.722	89
2021	Natural	E33	19	169	0.733	107
2021	Natural	E34	6	87	0.416	45
2021	Natural	E34	13	136	0.750	88
2021	Natural	E35	3	84	0.292	56
2021	Natural	E36	18	253	0.696	157
2021	Natural	E37	19	249	0.680	155

2021	Natural	E38	18	220	0.798	158
2021	Natural	E39	19	221	0.965	156
2021	Natural	E40	16	219	0.624	154

Ibis Field ID^{++} = the same bird, sampled over time, is indicated by the same Ibis Field ID number, within a given hal

S/N* = sample-to-negative ratio in bELISA: S/n values ≤ 0.7 are considered positive for antibodies to AIV

Ct$^{\#}$ = cycle threshold; samples with Ct ≤ 45 considered positive for AIV viral RNA

NS^{A} = NO SAMPLE: a sample of this type was not collected for a given bird or time-point

NA^{B} = NOT APPLICABLE: a test of the indicated type was not performed because no sample was collected

N/D^{C} = NOT DETECTED (rrt-PCR): IAV viral RNA was not detected in rrt-PCR with a cycle threshold ≤ 45

N/D^{D} = NOT DETECTED (virus isolation): IAV was not isolated from the sample when inoculated into embryonated

ological Screening

Ct[#] Value	Virus Isolation Result
N/D[C]	N/D[D]
N/D	N/D
N/D	N/D
N/D	N/D
N/D	N/D
N/D	N/D
N/D	N/D
N/D	N/D
N/D	N/D
N/D	N/D
N/D	N/D
N/D	N/D
N/D	N/D
N/D	N/D
N/D	N/D
N/D	N/D
N/D	N/D
N/D	N/D
N/D	N/D
N/D	N/D
N/D	N/D
N/D	N/D
N/D	N/D
N/D	N/D
N/D	N/D
N/D	N/D
N/D	N/D
N/D	N/D
N/D	N/D
N/D	N/D
N/D	N/D
N/D	N/D
N/D	N/D
N/D	N/D
N/D	N/D
N/D	N/D
N/D	N/D
N/D	N/D
N/D	N/D
N/D	N/D
N/D	N/D
N/D	N/D
N/D	N/D

N/D	N/D
N/D	N/D
N/D	N/D
N/D	N/D
N/D	N/D
N/D	N/D
N/D	N/D
N/D	N/D
N/D	N/D
N/D	N/D
N/D	N/D
N/D	N/D
N/D	N/D
N/D	N/D
N/D	N/D
N/D	N/D
N/D	N/D
N/D	N/D
N/D	N/D
N/D	N/D
N/D	N/D
N/D	N/D
N/D	N/D
N/D	N/D
N/D	N/D
N/D	N/D
N/D	N/D
N/D	N/D
N/D	N/D
N/D	N/D
N/D	N/D
N/D	N/D
N/D	N/D
N/D	N/D
N/D	N/D
N/D	N/D
N/D	N/D
N/D	N/D
N/D	N/D
N/D	N/D
N/D	N/D
N/D	N/D
N/D	N/D
N/D	N/D
N/D	N/D
N/D	N/D
N/D	N/D

N/D	N/D
43.98	N/D
N/D	N/D
N/D	N/D
NA	NA
N/D	N/D
N/D	N/D
N/D	N/D
N/D	N/D
N/D	N/D
N/D	N/D
N/D	N/D
N/D	N/D
N/D	N/D
N/D	N/D
N/D	N/D
N/D	N/D
N/D	N/D
N/D	N/D
N/D	N/D
N/D	N/D
N/D	N/D
N/D	N/D
N/D	N/D
N/D	N/D
N/D	N/D
N/D	N/D
N/D	N/D
N/D	N/D
N/D	N/D
N/D	N/D
N/D	N/D
N/D	N/D
N/D	N/D
N/D	N/D
N/D	N/D
N/D	N/D
N/D	N/D
N/D	N/D
NA	NA
N/D	N/D
N/D	N/D
N/D	N/D
N/D	N/D
N/D	N/D
N/D	N/D
N/D	N/D

N/D	N/D
N/D	N/D
N/D	N/D
N/D	N/D
N/D	N/D
N/D	N/D
N/D	N/D
N/D	N/D
N/D	N/D
N/D	N/D
N/D	N/D
N/D	N/D
N/D	N/D
N/D	N/D
N/D	N/D
N/D	N/D
N/D	N/D
N/D	N/D
N/D	N/D
N/D	N/D
N/D	N/D
N/D	N/D
N/D	N/D
N/D	N/D
N/D	N/D
N/D	N/D
N/D	N/D
N/D	N/D
N/D	N/D
N/D	N/D
N/D	N/D
N/D	N/D
N/D	N/D
N/D	N/D
N/D	N/D
N/D	N/D
N/D	N/D
N/D	N/D
N/D	N/D
N/D	N/D
N/D	N/D
N/D	N/D
N/D	N/D
N/D	N/D
NA	NA
N/D	N/D
N/D	N/D

N/D	N/D
N/D	N/D
N/D	N/D
N/D	N/D
N/D	N/D
N/D	N/D
N/D	N/D
N/D	N/D
N/D	N/D
N/D	N/D
N/D	N/D
N/D	N/D
NA	NA
N/D	N/D
N/D	N/D
N/D	N/D
N/D	N/D
N/D	N/D
N/D	N/D
N/D	N/D
N/D	N/D
N/D	N/D
N/D	N/D
N/D	N/D
N/D	N/D
N/D	N/D
N/D	N/D
N/D	N/D
NA	NA
N/D	N/D
NA	NA
N/D	N/D
N/D	N/D
NA	NA
NA	NA
NA	NA
N/D	N/D
NA	NA
NA	NA
N/D	N/D
N/D	N/D
N/D	N/D
NA	NA
N/D	N/D
N/D	N/D
N/D	N/D
N/D	N/D

N/D	N/D
N/D	N/D
N/D	N/D
N/D	N/D
N/D	N/D
33.98	N/D
N/D	N/D
N/D	N/D
N/D	N/D
N/D	N/D
N/D	N/D
N/D	N/D
N/D	N/D
N/D	N/D
N/D	N/D
N/D	N/D
N/D	N/D
N/D	N/D
N/D	N/D
N/D	N/D
N/D	N/D
N/D	N/D
N/D	N/D
N/D	N/D
N/D	N/D
N/D	N/D
N/D	N/D
N/D	N/D
N/D	N/D
N/D	N/D
N/D	N/D
N/D	N/D
N/D	N/D
N/D	N/D
N/D	N/D
N/D	N/D
N/D	N/D
N/D	N/D
N/D	N/D
N/D	N/D
N/D	N/D
N/D	N/D
N/D	N/D
N/D	N/D
N/D	N/D
N/D	N/D
N/D	N/D

N/D	N/D
N/D	N/D
N/D	N/D

bitat and within a given collection year.

chicken eggs
